# Evaluation of hypoxia-inducible factor-1α and urine non-transferrin-bound iron concentrations in cats with chronic kidney disease

**DOI:** 10.3389/fvets.2024.1482998

**Published:** 2024-12-19

**Authors:** Chien-Hui Chen, Wei-Li Hsu, Pei-Shiue Jason Tsai, Chun-Fu Lai, Meng-Ting Wu, Ya-Jane Lee

**Affiliations:** ^1^School of Veterinary Medicine, College of Bio-Resources and Agriculture, Institute of Veterinary Clinical Science, National Taiwan University, Taipei, Taiwan; ^2^Veterinary Hospital, College of Bio-Resources and Agriculture, National Taiwan University, Taipei, Taiwan; ^3^College of Veterinary Medicine, Graduate Institute of Microbiology and Public Health, National Chung-Hsing University, Taichung, Taiwan; ^4^School of Veterinary Medicine, College of Bio-Resources and Agriculture, Institute of Veterinary Medicine, National Taiwan University, Taipei, Taiwan; ^5^National Taiwan University Hospital, College of Medicine, National Taiwan University, Taipei, Taiwan; ^6^Department of Chemistry, College of Science, National Taiwan University, Taipei, Taiwan

**Keywords:** HIF, NTBI, CKD, noninvasive biomarkers, renal disease

## Abstract

**Introduction:**

Hypoxia-inducible factors (HIF) regulate gene transcription, which aids hypoxia adaptation while promoting renal fibrosis. Non-transferrin-bound iron (NTBI) is a catalytic form of iron that can lead to oxidative damage. However, NTBI in cat biofluids has rarely been evaluated.

**Aims:**

We assessed cat plasma and urine HIF-1α (pHIF-1α/uHIF-1α) concentrations and urine NTBI (uNTBI) concentrations to investigate their relationship with chronic kidney disease (CKD) severity.

**Methods:**

pHIF-1α and uHIF-1α concentrations were measured using commercial ELISA kits, while uNTBI concentrations were detected by inductively coupled plasma mass spectrometry.

**Results:**

Healthy cats (*n* = 35) and cats with CKD (*n* = 84) formed the study cohorts. pHIF-1α concentrations increased from 9.48 pg./mL (median) in the healthy cohort to 11.42 pg./mL in early-stage CKD cats but decreased to 8.50 pg./mL in late-stage CKD cats. uHIF-1α concentrations gradually decreased with a significant difference between the control group (44.61 pg./mL) and the late-stage CKD group (36.79 pg./mL, *p* < 0.001). Cats with proteinuria had significantly higher uNTBI concentrations (35.61 ppb) than non-proteinuric cats (25.13 ppb, *p* = 0.019). Finally, the concentrations of pHIF-1α and uHIF-1α were positively correlated independent of renal function.

**Conclusion and clinical importance:**

Overall, pHIF-1α and uHIF-1α concentrations are lower in advanced CKD cats, while uNTBI concentrations are significantly higher in proteinuric cats.

## 1 Introduction

Kidneys require large amounts of oxygen to maintain various essential functions that are necessary for survival (1). During hypoxia, kidneys must adapt to a low oxygen level while still maintaining these vital functions (1). Hypoxia-inducible factor (HIF), a transcription factor regulating the expression of many genes, including erythropoietin (EPO), is crucial to the kidneys’ defense against hypoxia (1). However, although HIF can stimulate the expression of genes that help renal cells adapt to hypoxia, it is also associated with fibrosis and the progression of nephropathy (1). Studies have also demonstrated a positive correlation between urine HIF concentrations and chronic kidney disease (CKD) severity in humans (2, 3). Nonetheless, experimental research has revealed that the level of HIF in renal tissue does not affect the development of kidney fibrosis (4). These results mean that the role of HIF in CKD progression remains controversial. In cats, renal tissue HIF expression is significantly elevated during CKD (5) and is positively correlated with the cat’s fibrosis status (6). Nevertheless, research on HIF concentrations in biofluids from CKD cats is lacking.

Iron is an essential substrate and is involved in several physical mechanisms including oxygen transport, especially when cells have a high metabolic demand, for example, renal cells (7). In CKD, impaired renal function leads to an increased loss of iron and decreased EPO production; this subsequently leads to decreased levels of systemic iron and EPO, which ultimately results in iron deficiency and anemia (7). As a result, iron administration is usually prescribed in such circumstances (8). However, this treatment may also elevate the risk of iron-induced kidney injury, which will occur in the absence of a body excretion process for an iron overload (9). Non-transferrin-bound iron (NTBI), a catalytic form of iron that has been linked to oxidative damage, is detectable when iron overload is present (7). Several studies have investigated serum NTBI concentrations in patients under chelation therapy or having been prescribed iron supplementation (10) as well as in dialysis patients (11, 12). Our team has previously measured the presence of increased serum NTBI concentrations in CKD cats (in press). While urine NTBI concentrations are known to be elevated during iron overload and in the presence of impaired renal function in humans (7, 9), nevertheless similar research is not available in the veterinary medicine literature.

The contribution of HIF to iron homeostasis has been demonstrated (13). The activation of HIF is thought to regulate several iron-handling proteins and EPO, and this can then result in enhanced iron utilization and erythropoiesis (13). Taking this into account, HIF stabilizers have been identified as a new treatment for CKD anemia (14). Human studies have demonstrated an improvement in anemia together with better iron utilization when CKD patients receive HIF stabilizers (15, 16); unfortunately, NTBI concentrations were not evaluated in these previous studies. Thus, in veterinary medicine, although HIF stabilizers might be beneficial to cats with CKD when they are assessed by a change in a cat’s hematocrit (17), investigations targeting NTBI concentrations have remained notably absent. In addition, due to a lack of knowledge on HIF concentrations at the different stages of CKD, there is a need for further research in these areas to determine whether the administration of HIF stabilizers does elevate HIF concentrations in CKD cats, which could potentially cause adverse effect on the kidneys.

The present study aims to assess HIF-1α and NTBI concentrations in the biofluids of CKD cats. The objectives are as follows: (1) to evaluate the concentrations of HIF-1α in the plasma and urine (pHIF-1α/uHIF-1α), as well as the concentrations of NTBI in urine (uNTBI), using both healthy cats and cats with CKD; (2) to investigate the correlations between the concentrations of HIF-1α and NTBI in biofluids. In addition to the above direct comparisons, a similar comparison targeting various clinical variables was also carried out using the same feline cohorts; (3) to elucidate the relationship between pHIF-1α and uHIF-1α concentrations.

## 2 Materials and methods

### 2.1 Animals

Cats attending the National Taiwan University Veterinary Hospital (Taipei, Taiwan) for health assessment or CKD control were recruited from December 2020 to August 2023.

Cats simultaneously satisfying the following standards were classified into the control cohort: (1) age > 1 year old regardless of gender and breed; (2) the lack of any concurrent disease and any medical therapy other than parasitic prophylaxis using commercial products; (3) the absence of a past history of any renal, neoplastic, endocrine, neurological, or cardiovascular system disease; (4) evidence available of their clinical and physical examination results, appraised by a veterinarian, which were normal; (5) a normal cell blood count (CBC) (Exigo, United States; ProCyte Dx, IDEXX, United States) together with a full biochemistry, including albumin, total protein, glucose, alkaline phosphatase (ALKP), alanine aminotransferase (ALT), aspartate transaminase (AST), blood urea nitrogen (BUN, <35 mg/dL), plasma creatinine (< 2.0 mg/dL) (Vitros 350 Chemistry System, Ortho-Clinical Diagnostics, United States), plasma symmetric dimethylarginine (SDMA,<14 μg/dL), total thyroxine (Catalyst One, IDEXX, United States), sodium, potassium, and chloride (cobas b 121, Roche Diagnostics Ltd., Taiwan); (6) the presence of normal urinalysis, including urine specific gravity (USG > 1.035), pH, dipstick, sediments, and urine protein-to-creatinine ratio (UPC < 0.2, Catalyst One, IDEXX, United States); (7) no abnormalities detectable by abdominal X-ray and ultrasonography.

Cats that met any one of the following criteria were selected to form the CKD cohort: (1) persistent azotemia identified by a plasma creatinine concentration > 2.0 mg/dL, or a plasma SDMA concentration persistently >14 μg/dL on three occasions at least 2 weeks apart, without prerenal and postrenal causes; (2) a USG level < 1.035 with a plasma creatinine concentration > 2.0 mg/dL, or the presence of persistent proteinuria as indicated by a UPC > 0.4 on three occasions at least 2 weeks apart, in the absence of active sediments; (3) abnormal image findings, such as the presence of renal calculi, cysts, infarcts, or alteration in the size, shape, and echogenicity of the kidneys. Subsequently, cats with CKD were then grouped based on International Renal Interest Society (IRIS) staging.

Cats presenting with any one of the following conditions, namely acute kidney injury (AKI), urinary tract infection (UTI), feline lower urinary tract disease (FLUTD), neoplastic diseases, endocrine disease, and those receiving iron supplements or EPO-stimulating agents, were excluded.

In the present study, anemia was defined as a hematocrit <25.0%. Cats with systolic arterial blood pressure (SAP) > 160 mmHg measured over 1 to 2 weeks were classified as hypertensive, while proteinuria was present if UPC > 0.4 on three occasions at least 2 weeks apart in the absence of active sediments.

### 2.2 Sample collection and storage

Blood samples were collected from either the cephalic or inner saphenous veins. The samples were transferred into plasma separation tubes containing lithium heparin for plasma collection. The tubes were then centrifuged at 12000 g for 45 s, and further centrifugation was carried out based on the required separating conditions. The plasma was stored at -80°C until further examination. Samples with hemolysis were discarded.

Urine samples were harvested by either cystocentesis or free catch. The samples were centrifuged at 1,500 g for 3 min, and the supernatants were kept at −80°C until utilization.

### 2.3 Measurement of HIF-1α concentrations by ELISA

The concentrations of pHIF-1α and uHIF-1α were assessed using commercial enzyme-linked immunosorbent assay (ELISA) kits that had been validated for cats (#MBS097218, MyBioSource, United States). The reagents of the kits and the processed samples were thawed to room temperature before use. The ELISA assays were carried out as indicated by the manual. All the samples were tested in duplicate. The validation data is provided in Supplementary Table S1. Of note, measured concentrations of less than 6.25 pg./mL (the limit of detection) are presented as 3.125 pg./mL.

### 2.4 Measurement of NTBI concentrations by ICP-MS

Owing to the sensitivity needed for iron detection, the inductively coupled plasma mass spectrometry (ICP-MS) method was chosen for the biofluid NTBI concentration analysis. uNTBI concentrations were detected by Agilent 7700e (Agilent Technologies, California, United States) ICP-MS. The method for sample preparation was modified from a previous study (18). A more comprehensive description is given below, and the validation data is presented in Supplementary Tables S2–S8 and Supplementary Figure S1.

### 2.5 Chemicals and reagents

ULTREX^®^ Ultrapure grade concentrated nitric acid (67.0–70.0% HNO_3_) was purchased from J.T. Baker Chemical Co. (Pennsylvania, United States), while VWR Chemicals BDH^®^ Magnesium chloride hexahydrate 99.0–102.0% ACS was obtained from VWR International (Pennsylvania, United States). Certipur^®^ ICP Standard solution of iron (1,000 ppm), Amicon^®^ Ultra-0.5 mL filters (30 kilodaltons, kDa), and Millex-GV syringe filter unit (0.22 μm, PVDF, 33 mm) were acquired from Merck KGaA (Darmstadt, Germany). MetalFree^®^ 15 mL/50 mL centrifuge tubes were procured from Labcon (California, United States). Analytical grade reagents and high-purity 18.2 MegΩ cm deionized water, were obtained from Purity-SP (LotunTechnic, Taipei, Taiwan), Disposable 200 μL/1,000 μL sterile racked filtered tips (SSIbio, California, United States), and 60 μL/150 μL microcentrifuge tubes (Eppendorf, Hamburg, Germany) were also used in the experiments. Operators prepared all solutions in fume hoods with powder-free gloves and masks. The glass pipette, volumetric flask, and beakers were cleaned with an acid bath and by ultrasonic cleaning, which was followed by rinsing with deionized water and subsequent drying.

### 2.6 Instrumentation

The Agilent 7700e (Agilent Technologies, California, United States) ICP-MS system was used for analysis. A KUBOTA Model 5,922 (KUBOTA, Tokyo, Japan) universal refrigerated centrifuge was employed to prepare the serum ultrafiltrates.

### 2.7 Stock solutions and calibration standards

The 67.0–70.0% nitric acid was diluted with deionized water to achieve a 2% nitric acid solution. Magnesium chloride hexahydrate was dissolved in the 2% nitric acid to obtain the dilution agent (0.0049 M MgCl_2_ with 2% HNO_3_), and this was then used in the subsequent procedures. The standard solution of iron (1,000 ppm) was diluted with the dilution agent mentioned above to obtain the stock solution of iron (100 ng/mL). The stock solution was then diluted with the dilution agent to generate calibration standards at various concentrations (from 5 to 50 ng/mL). The stock solution and the calibration standards were kept at 4°C and renewed monthly.

### 2.8 Sample preparation using the ultrafiltration method

Urine samples were frozen at -80°C and then thawed naturally to room temperature before further processing. The magnesium chloride hexahydrate was dissolved in the ultrapure water to obtain a 0.05 M magnesium chloride solution. Next 400 μL of the solution was combined with 100 μL of the urine sample in a clean 1.5 mL microcentrifuge tube and incubated at room temperature for 20 min. An Amicon^®^ filter was then filled with 400 μL of the ultrapure water and centrifuged at 14,000 g for 10 min to rinse the filter before use. Each sample, processed as described above, was loaded into a rinsed Amicon^®^ filter. Groups of filters were then centrifuged at 14,000 g for 20 min to separate the free iron formed as a chloride complex. Next, 2.85 mL of 2% nitric acid was added to 15 mL metal-free centrifuge tubes, and 0.4 mL of each centrifuged solution containing the free iron was then transferred into these tubes. These processed samples were finally filtered through Millex-GV filters into new 15 mL metal-free centrifuge tubes and stored at 4°C until the subsequent ICP-MS analysis on the next day.

### 2.9 ICP-MS operating conditions

After considering the levels of the different elements and their isotopes present in nature, ^57^Fe was selected for the NTBI concentration detection. The operating conditions used for ICP-MS analysis are summarized in Supplementary Table S9.

The ICP-MS was conducted as follows. In brief, the samples were sucked by the peristaltic pump, aerosolized by the nebulizer with argon gas, and then entered the spray chamber, which would filter out large aerosol droplets. The samples were then desolvated, vaporized, atomized, and ionized after leaving the spray chamber to reach the high-temperature plasma. The iron ion beam then passed through the interface region and was guided by the ion optics system to reach the mass analyzer, which detected the signals of the ions and finally converted them into the final concentrations by the external calibration method. To ensure the integrity and reliability of the system, meticulous pre-operational maintenance was conducted. Each time, before operation, the cones were cleaned, and the instrumental settings of ICP-MS were optimized using a tuning solution consisting of 1 μg/L of Ce, Co, Li, Mg, Ti, and Y to establish system reliability.

### 2.10 Statistical analysis

Data was analyzed using R 4.3.3 with RStudio 2023.12.1 + 402 for macOS. Due to the limited sample size of the study cohorts, it was challenging to achieve a normal distribution for the continuous data. Thus, the distribution of the continuous variables is presented as medians and interquartile ranges (IQRs). Fisher’s exact test or the Chi-Square test was conducted to assess whether the categorical variables show statistical significance among groups, while the Mann Whitney U test or the Kruskal-Wallis test, with the Bonferroni test as the *post-hoc* analysis, was used for the continuous variables. Correlations between continuous variables were evaluated using Spearman’s rank test followed by partial correlation to allow further investigation. Likewise, simple linear regression as well as further assessment by multiple linear regression were also conducted. *p*-value <0.05 is significant.

## 3 Results

### 3.1 Animals

A total of 119 cats were enrolled in the study, with 35 healthy cats in the healthy cohort and 84 cats with CKD in the CKD cohort. Among the cats with CKD, 8 were stage 1, 44 were stage 2, 28 were stage 3, and 4 were stage 4. To enhance the statistical analysis, cats in the CKD stage 1 and 2 groups were combined into the early-stage CKD group (*n* = 52), while the CKD stage 3 and 4 groups were combined into the late-stage CKD group (n = 32). Furthermore, 15 CKD cats showed anemia, 13 had hypertension, and 19 suffered from proteinuria. The median age of the 119 cats was 7.0 years (IQRs: 3.3–12.5 years). Most cats were castrated males (*n* = 59) and spayed females (*n* = 52). Furthermore, the domestic short hair (n = 88) was the predominant breed, followed by the American short hair (*n* = 6), the British short hair (*n* = 4), the Exotic short hair (*n* = 4), the Ragdoll (*n* = 4), the Bengal (*n* = 2), the Chinchilla (*n* = 2), the Persian (*n* = 2), the Russian blue (*n* = 2), the Scottish fold (*n* = 2), and one each of the British long hair, the Munchkin, and the Siamese. Table 1 presents the demographic and clinicopathological data of cats that formed the two cohorts in the present study.

**Table 1:** The demographic and clinicopathologic data of cats in the present study.

Variable	Control	n	Early CKD	n	Late CKD	n	*p*
Demographic data							
Age (year)	6.00 (2.50, 8.00)	35	6.00 (2.00, 11.25)	52	12.50 (7.75, 16.25)^AB^	32	**< 0.001**
Female	20 (57.1)	35	21 (40.4)	52	14 (43.8)	32	0.329
Neutered	34 (97.1)	35	48 (92.3)	52	29 (90.6)	32	0.811
Domestic short hair breed	27 (77.1)	35	38 (73.1)	52	23 (71.9)	32	0.910
Body weight (kg)	5.04 (4.66, 5.56)	35	4.44 (3.83, 5.39)	52	4.55 (3.56, 5.09)^A^	32	**0.034**
Body condition score (/9)	6.0 (5.0, 7.0)	35	5.0 (5.0, 6.0)^A^	52	5.0 (4.0, 6.0)^A^	32	**0.001**
SAP (mmHg)	147.0 (126.0, 159.5)	18	136.0 (120.5, 147.0)	27	149.0 (135.0, 154.0)	23	0.244
Antihypertensive drugs	0 (0)	35	4 (7.7)	52	7 (21.9)	32	**< 0.001**
Phosphorous binders	0 (0)	35	2 (3.8)	52	14 (43.8)	32	**< 0.001**
Renal prescription diet	0 (0)	35	8 (15.4)	52	20 (62.5)	32	**< 0.001**
CKD complications							
Anemia	0 (0)	35	5 (9.6)	52	10 (31.2)	32	**< 0.001**
Hypertension	0 (0)	35	5 (9.6)	52	8 (25)	32	**0.006**
Proteinuria	0 (0)	35	9 (17.3)	52	10 (31.2)	32	**< 0.001**
Complete blood count							
Hemoglobin (g/dL)	14.80 (13.60, 15.55)	35	13.70 (12.30, 15.57)	50	12.15 (9.75, 13.68)^AB^	28	**< 0.001**
Hematocrit (%)	43.90 (40.15, 46.75)	35	40.65 (36.60, 45.92)	50	36.05 (28.77, 40.27)^AB^	28	**< 0.001**
RBC (10^6^/uL)	9.03 (8.50, 9.86)	35	8.62 (7.57, 9.97)	50	8.02 (6.06, 8.67)^A^	28	**0.004**
WBC (10^9^/L)	7,700 (6,950, 8,850)	35	7,750 (6,100, 10,275)	50	9,000 (6,425, 10,850)	28	0.468
Biochemistry							
Plasma SDMA (ug/dL)	7.0 (4.0, 10.0)	35	11.0 (8.0, 19.0)^A^	45	22.0 (15.0, 25.0)^AB^	23	**< 0.001**
BUN (mg/dL)	21.0 (19.5, 23.0)	35	27.0 (21.0, 31.0)^A^	52	40.5 (36.8, 50.5)^AB^	32	**< 0.001**
Plasma creatinine (mg/dL)	1.70 (1.40, 1.85)	35	2.00 (1.70, 2.50)^A^	52	3.50 (3.20, 4.03)^AB^	32	**< 0.001**
Na (mmol/L)	155.2 (153.9, 156.3)	35	155.3 (153.3, 156.7)	49	154.8 (153.1, 156.8)	30	0.873
K (mmol/L)	3.73 (3.39, 4.06)	35	3.82 (3.55, 4.07)	49	4.23 (3.98, 4.53)^AB^	30	**< 0.001**
Cl (mmol/L)	116.4 (114.7, 117.7)	35	116.3 (114.4, 117.7)	49	117.2 (115.5, 118.2)	30	0.461
Urinalysis							
USG	1.049 (1.044, 1.053)	35	1.024 (1.013, 1.042)^A^	52	1.012 (1.010, 1.012)^AB^	32	**< 0.001**
pH	6.34 (6.05, 6.70)	35	6.03 (5.85, 6.59)	52	5.66 (5.43, 6.32)^AB^	32	**< 0.001**
UPC	0.02 (0.01, 0.04)	35	0.06 (0.02, 0.16)^A^	52	0.15 (0.07, 0.55)^AB^	32	**< 0.001**
Urine creatinine (mg/dL)	444.6 (348.0, 493.9)	35	169.0 (113.2, 328.2)	52	108.0 (85.8, 123.5)	32	**< 0.001**

Clinical data of significance, physical examination results, CBC, biochemistry, and urinalysis are presented in the table. Fisher’s exact test and Kruskal-Wallis test with the Bonferroni test as the post-hoc correction was used. Categorial and continuous data were demonstrated as number (%) and median (IQRs), respectively. SAP, systolic arterial blood pressure; RBC, red blood cells count; WBC, white blood cells count; BUN, blood urea nitrogen; SDMA, symmetric dimethylarginine; USG, urine specific gravity; UPC, urine protein-to-creatinine ratio. Early CKD: IRIS stage 1 and 2 CKD. Late CKD: IRIS stage 3 and 4 CKD. Antihypertensive drugs: including angiotensin converting enzyme inhibitors, angiotensin receptor blockers, and calcium channel blockers. Phosphorous binders: including lanthanum carbonate, ferric citrate, and sucralfate. *p*-value < 0.05 is significant; the significant *p*-values are marked in bold. ^A^Achieved significance against the control group. ^B^Achieved significance against the early CKD group.

### 3.2 pHIF-1α concentrations

**Figure 1:**
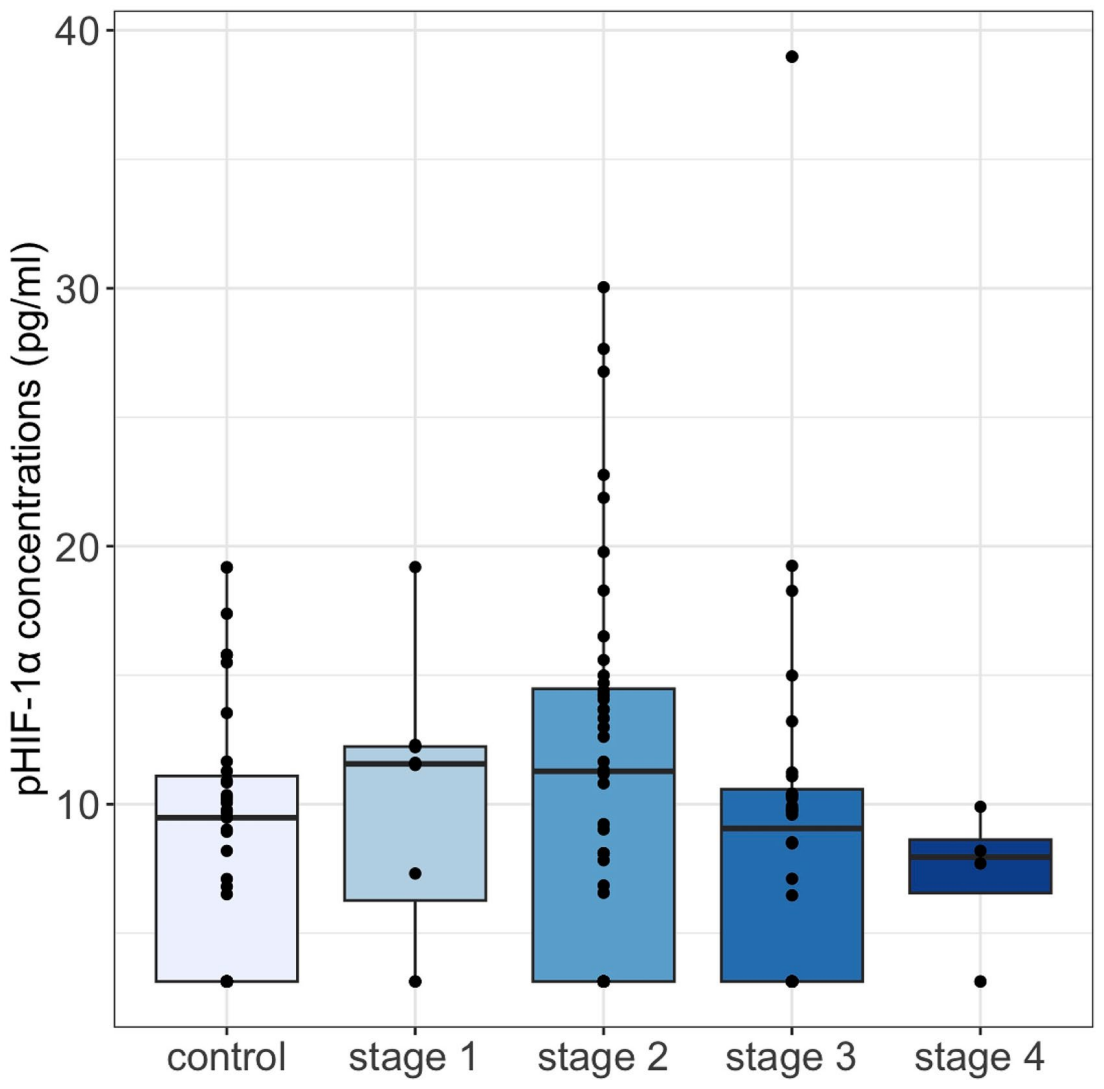
Plasma HIF-1α concentrations were compared across control cats and cats with IRIS CKD stages 1–4 using the Kruskal-Wallis test, with the Bonferroni test for post-hoc analysis. No significant differences were observed. Median pHIF-1α concentrations were highest in cats with stage 1 CKD, showing an increase in early-stage CKD followed by a decline as CKD severity progressed. pHIF-1α, plasma hypoxia-inducible factor 1α.

Among the 119 cats, 36 cats (including 11 in the healthy cohort, 2 in the stage 1 group, 12 in the stage 2 group, 10 in the stage 3 group, and 1 in the stage 4 group) had pHIF-1α concentrations of less than 6.25 pg./mL. Overall, pHIF-1α concentrations (median [IQRs]) were numerically higher in the healthy cohort (9.48 [3.13, 11.09] pg./ml) than in early-stage CKD cats (11.42 [3.13, 14.29] pg./ml), but pHIF-1α concentrations then decreased when the cats progressed to late-stage CKD (8.50 [3.13, 10.35] pg./ml). Specifically, cats with stage 1 CKD had the highest pHIF-1α concentrations among all the groups (Figure 1).

### 3.3 uHIF-1α concentrations

**Figure 2:**
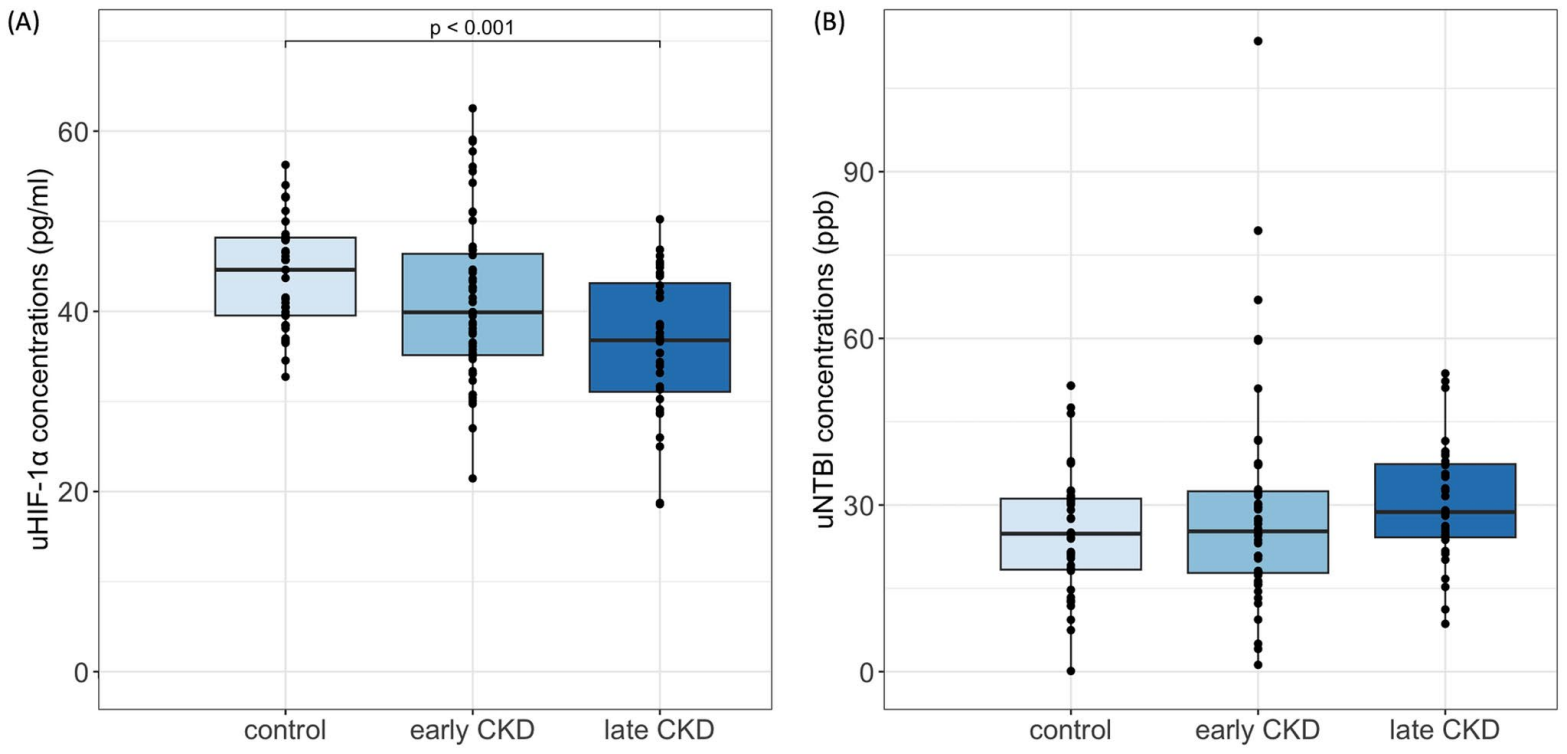
uHIF-1α and uNTBI concentrations in cats. **(A)** uHIF-1α concentrations significantly decreased in late CKD (IRIS stages 3–4) compared to controls (*p* < 0.001), Kruskal-Wallis with Bonferroni *post hoc*. **(B)** uNTBI concentrations showed a nonsignificant increasing trend with CKD progression. uHIF-1α, urinary hypoxia-inducible factor 1 α; uNTBI, urinary non-transferrin bound iron. Early CKD, IRIS stages 1–2; Late CKD, IRIS stages 3–4.

In contrast, the concentrations of uHIF-1α in cats with early-stage CKD (39.90 [35.14, 46.38] pg./ml) were numerically lower than the concentrations of the healthy cohort (44.61 [39.54, 48.18] pg./ml) and the concentrations then became even lower when the cats progressed to late-stage CKD (36.79 [31.07, 43.13] pg./ml). Moreover, a significant difference was present between the control and late-stage CKD groups (*p* < 0.001) (Figure 2A).

### 3.4 uNTBI concentrations

On the other hand, uNTBI concentrations progressively increased in CKD cats as compared to the control cats. Among the various groupings, the control cohort (24.84 [18.37, 31.15] ppb) had the lowest uNTBI concentrations, and the concentrations then increased for the early-stage CKD group (25.27 [17.78, 32.47] ppb) and then increased again for the late-stage CKD group (28.75 [24.20, 37.37] ppb). Despite there being no significant difference in these concentrations, the variation in uNTBI concentrations does represent an elevated trend as CKD severity increases (Figure 2B).

### 3.5 Spearman and partial correlation

Analysis of the Spearman’s rank test demonstrated a significantly positive correlation of pHIF-1α concentrations with uHIF-1α concentrations (rho = 0.54, *p* < 0.001) and with systolic arterial blood pressure (rho = 0.26, *p* = 0.032) (Table 2). Furthermore, a significant correlation between pHIF-1α and uHIF-1α concentrations remained after the data underwent the partial correlation test, taking plasma creatinine (rho = 0.48, *p* < 0.001) and urine creatinine (rho = 0.46, *p* < 0.001) as confounders.

**Table 2:** The Spearman’s rank test of HIF-1α and NTBI.

	pHIF-1α		uHIF-1α		uNTBI	
	Rho	*p*	Rho	*p*	Rho	*p*
pHIF-1α	–	–	0.54	< 0.001	−0.06	0.498
uHIF-1α	0.54	**< 0.001**	–	–	−0.15	0.095
uNTBI	−0.06	0.498	−0.15	0.095	–	–
age	−0.02	0.860	−0.15	0.096	0.09	0.356
SAP	0.26	**0.032**	−0.04	0.737	< 0.01	0.968
Hemoglobin	0.16	0.094	0.22	**0.021**	−0.10	0.298
Hematocrit	0.15	0.119	0.24	**0.009**	−0.11	0.262
RBC	0.08	0.407	0.12	0.198	0.01	0.903
BUN	−0.06	0.526	−0.29	**0.001**	0.11	0.239
Plasma creatinine	−0.08	0.379	−0.31	**< 0.001**	0.17	0.071
Plasma SDMA	−0.02	0.849	−0.31	**0.002**	0.13	0.180
K	−0.09	0.354	−0.18	0.052	0.08	0.420
USG	0.14	0.140	0.38	**< 0.001**	−0.16	0.082
UPC	−0.04	0.666	−0.28	**0.002**	0.19	**0.038**

The correlations between biomarkers of interest and other clinical data are presented in the table. pHIF-1α, plasma HIF-1α; uHIF-1α, urine HIF-1α; uNTBI, urine NTBI; SAP, systolic arterial blood pressure; RBC, red blood cells count; BUN, blood urea nitrogen; SDMA, symmetric dimethylarginine; USG, urine specific gravity; UPC, urine protein-to-creatinine ratio. *p*-value < 0.05 is significant; the significant p-values are marked in bold.

Compared to pHIF-1α concentrations, the uHIF-1α concentrations exhibited correlations with a broader range of clinical variables (Table 2). uHIF-1α concentrations showed significant negative correlations with BUN (rho = −0.29, *p* = 0.001), with plasma creatinine (rho = −0.31, *p* < 0.001), with plasma SDMA (rho = −0.31, *p* = 0.002), and with UPC (rho = −0.28, *p* = 0.002). On the other hand, the correlations with hemoglobin (rho = 0.22, *p* = 0.021), with hematocrit (rho = 0.24, *p* = 0.009), and with USG (rho = 0.38, *p* < 0.001) were significantly positive. In terms of uNTBI concentrations, a significant positive correlation was only noticed when linked to UPC (rho = 0.19, *p* = 0.038) (Table 2).

### 3.6 Simple and multiple linear regression

Using linear regression, the pHIF-1α concentrations increased significantly in parallel with the elevation of uHIF-1α concentrations or with red blood cell counts (RBCs). As a further evaluation, variables with *p*-values less than 0.05 were investigated using stepwise regression. Finally, uHIF-1α concentrations and RBCs remained significantly correlated with pHIF-1α concentrations even after adjustment using multiple regression analysis (Table 3). Various other variables demonstrated significant correlations with uHIF-1α concentrations. Specifically, in addition to pHIF-1α concentrations, variables, including hemoglobin, hematocrit, and USG, showed significant positive correlations. On the other hand, renal indices and UPC revealed significant negative correlations. Subsequently, the variables with significance were analyzed using stepwise regression (BUN, plasma SDMA, and USG excepted); pHIF-1α concentrations, hematocrit, and plasma creatinine remained significant (Table 3). As for uNTBI concentrations, RBCs was the sole variable that shared a significant positive correlation when using the simple linear regression model (Table 3).

**Table 3:** The simple linear and stepwise regression results of HIF-1α and NTBI.

	pHIF-1α				uHIF-1α				uNTBI			
	Simple		Stepwise		Simple		Stepwise		Simple		Stepwise	
	Coeff	*p*	Coeff	*p*	Coeff	*p*	Coeff	*p*	Coeff	*p*	Coeff	*p*
pHIF-1α	–	–	–	–	0.63	**< 0.001**	0.63	**< 0.001**	−0.22	0.308	–	–
uHIF–1α	0.37	**< 0.001**	0.37	**< 0.001**	–	–	–	–	−0.29	0.082	–	–
uNTBI	−0.04	0.308	–	–	−0.09	0.082	–	–	–	–	–	–
Age	−0.04	0.739	–	–	−0.28	0.063	–	–	0.16	0.568	–	–
SAP	0.05	0.201	–	–	−0.05	0.337	–	–	−0.05	0.604	–	–
Hemoglobin	0.50	0.053	–	–	0.85	**0.010**	−0.95	0.109	−0.17	0.780	–	–
Hematocrit	0.05	0.575	–	–	0.29	**0.005**	0.41	**0.024**	−0.33	0.086	–	–
RBC	0.32	**0.006**	0.32	**0.002**	−0.01	0.939	–	–	0.82	**0.002**	–	–
BUN	−0.03	0.346	–	–	−0.10	**0.006**	–	–	0.05	0.459	–	–
Plasma Creatinine	−0.40	0.357	–	–	−1.90	**< 0.001**	−1.33	**0.021**	1.33	0.196	–	–
Plasma SDMA	−0.07	0.294	–	–	−0.24	**0.002**	–	–	0.15	0.243	–	–
K	−0.94	0.459	–	–	−2.87	0.078	–	–	0.81	0.788	–	–
USG	37.30	0.242	–	–	176.27	**< 0.001**	–	–	−105.90	0.158	–	–
UPC	−0.95	0.149	–	–	−1.88	**0.027**	<0.01	0.100	2.18	0.160	–	–

Variables were analyzed using simple linear regression first. Those with p-values less than 0.05 (BUN, plasma SDMA, and USG excepted) were later selected and assessed using stepwise regression. pHIF-1α, plasma HIF-1α; uHIF-1α, urine HIF-1α; uNTBI, urine NTBI; SAP, systolic arterial blood pressure; RBC, red blood cells count; BUN, blood urea nitrogen; SDMA, symmetric dimethylarginine; USG, urine specific gravity; UPC, urine protein-to-creatinine ratio. *p*-value < 0.05 is significant; the significant p-values are marked in bold.

### 3.7 Subgroup evaluation

**Figure 3:**
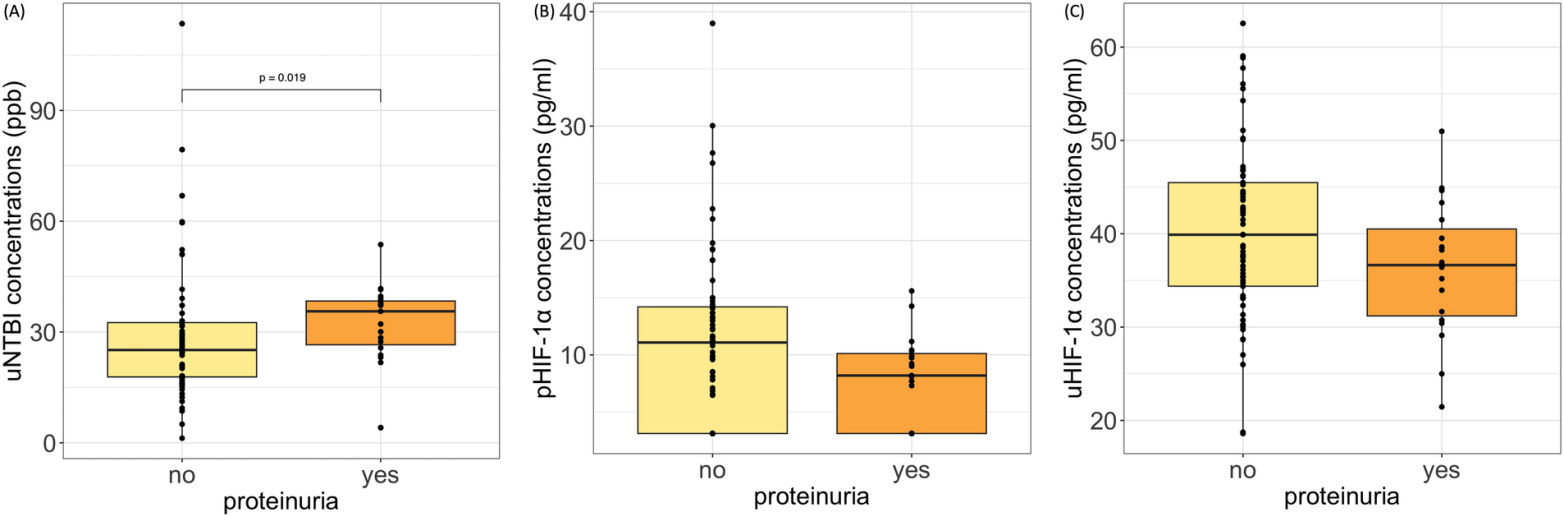
pHIF-1α, uHIF-1α, and uNTBI concentrations in cats with and without proteinuria. **(A)** uNTBI concentrations were significantly higher in proteinuric cats (*p* = 0.019, Mann–Whitney U test). **(B,C)** No significant differences were found in pHIF-1α or uHIF-1α concentrations. Proteinuria: urine protein-to-creatinine ratio (UPC) > 0.4; Non-proteinuria: UPC ≤ 0.4. pHIF-1α: plasma hypoxia-inducible factor 1α, uHIF-1α: urinary hypoxia-inducible factor 1 α, and uNTBI: urinary non-transferrin bound iron.

We further evaluated whether these biomarkers are correlated with significant differences between the various groups based on CKD complications. Cats with proteinuria presented with significantly higher uNTBI concentrations (35.61 [26.55, 38.39] ppb) than non-proteinuric cats (25.13 [17.85, 32.54] ppb, *p* = 0.019) (Figure 3A). Although the proteinuric cats had lower pHIF-1α and uHIF-1α concentrations, no significant differences were noted (Figures 3B,C). Moreover, in cats suffering from anemia or hypertension, their pHIF-1α, uHIF-1α, and uNTBI concentrations showed no significant differences compared to the CKD cats without these complications (data not shown).

## 4 Discussion

To our knowledge, this is the first study evaluating the concentrations of HIF-1α in the biofluids of cats, and it also establishes a method that successfully measures uNTBI concentrations in cats by ICP-MS. Furthermore, a comparative analysis was conducted between healthy control cats and cats with CKD for these markers. Human studies have identified that urine HIF-1α primarily originates from renal cells (3) and that urine NTBI is associated with impaired renal function (9). Our findings in the present study add to this by contributing to a better understanding of feline CKD.

In the present study, although not statistically significant, plasma HIF-1α concentrations showed a trend of being higher during the early stages of CKD (stages 1–2). However, these concentrations then gradually declined as CKD advanced to its later stages (stages 3–4) (Figure 1). In type 2 diabetes patients with different urine albumin-to-creatinine ratios (UACR), serum HIF-1α concentrations were significantly elevated, along with significantly increased concentrations of serum BUN and serum creatinine (19). Furthermore, previously, significant positive correlations between the serum concentrations of HIF-1α and BUN and between the serum concentrations of HIF-1α and serum creatinine have been found (19). Additionally, regarding HIF-1α mRNA concentrations in serum, significantly higher values have been noted in patients with autosomal dominant polycystic kidney disease (20). Since these patients were mostly classified as early-stage CKD (21), these results might help to explain the elevated pHIF-1α concentrations in our early-stage CKD cats. On the other hand, research has revealed that indoxyl sulfate, a uremic toxin, is able to suppress nuclear HIF-1α protein accumulation in HepG2 cells (22). Other research using hypoxia conditions has shown that uremic mesenchymal stem cells express less HIF-1α protein than control cells in normal levels of oxygen (23). These results indicate that uremia would seem to impair HIF-1α protein expression in various cells and that this might be correlated with the progressive reduction of pHIF-1α concentrations found in late-stage CKD cats during the present study (Figure 1).

Cats in the present study presented with a reduction in uHIF-1α concentrations as CKD progressed (Figure 2), and a significant negative correlation between uHIF-1α and various renal indices was noticed (Tables 2, 3). This is different from previous studies in humans, where significantly higher concentrations of urine HIF-1α protein were present in lupus nephritis patients (2); furthermore, there were also significantly higher concentrations of urine HIF-1α mRNA in CKD patients (3). Regarding feline studies, expression of the renal HIF-1α gene has been shown to be significantly increased in cats with naturally occurring CKD (5). However, lupus nephritis in humans is a glomerular disease (24), and feline CKD is generally a tubulointerstitial disease (25). This variation in the nature of the pathogenesis might be the cause of the above departure between cats and humans. Renal tissue volume is known to decline as CKD advances (26). While the glomerular filtration barrier typically restricts proteins larger than 40 kDa (27), and the HIF-1α protein has a molecular weight of approximately 120 kDa (28), the presence of HIF-1α in urine likely indicates renal dysfunction. This reduction in uHIF-1α concentrations during feline CKD may reflect increased HIF-1α accumulation in renal tissue as kidney damage advances. Overall, uHIF-1α shows potential as a biomarker for assessing CKD in cats.

It has been hypothesized that uNTBI concentrations would be higher in CKD cats as their renal tubular function becomes impaired. In line with this hypothesis, the present study revealed a rise in uNTBI concentrations (Figure 2). This result is consistent with previous studies on human patients with nephrotic syndrome and tubulopathy (7). Thus, we suggest that a rise in uNTBI concentrations can be used as a potential biomarker when investigating renal function in CKD cats.

The concentrations of pHIF-1α and uHIF-1α show a significant positive correlation even after undergoing the partial correlation test and adjusting for plasma creatinine or urine creatinine. Similarly, serum and urine HIF-1α mRNA concentrations moved in the same direction as that occurring in nephropathy humans, despite correlation tests not being conducted during this earlier research (20). The result of the partial correlation test in our study shows that there is a correlation between pHIF-1α and uHIF-1α concentrations that is independent of renal function. This might explain the inconsistent variations in pHIF-1α and uHIF-1α concentrations in our CKD cohort across the various stages of the disease.

A renal biopsy study on human patients has proposed that renal tubular iron deposition might play a detrimental role in CKD progression (29). Although studies targeting the correlation between biofluid NTBI concentrations and CKD progression are rare, iron-induced kidney injury and the relationship between kidney injury and increased urine iron concentrations should not be underestimated (7, 9). Elevated urine iron loss has been observed in CKD humans with proteinuria (30–32). Consistent with this, in the present study, proteinuria in cats was found to be related to a significant rise in uNTBI concentrations. These findings seem to suggest a potential relationship between urinary NTBI concentrations and CKD progression, as proteinuria is recognized as a negative prognostic factor (33). However, further investigation of iron dysregulation and CKD prognosis is warranted.

Anemia is a common complication of CKD, and iron supplements can serve as a treatment (8). Several studies have investigated the relationship between NTBI, iron supplements, and CKD. Serum NTBI has been identified in 22% of human end-stage renal disease patients treated with intravenous iron supplements for anemia (10). Other research has demonstrated that nearly 20% of human dialysis patients show significantly increased plasma NTBI concentrations after 1 week or more of iron administration (11). Therefore, cats that were receiving iron supplementation were excluded from the present study. In addition, studies related to the correlation between EPO-stimulating agents and HIF-1α concentrations in biofluids are few. Since EPO is the target gene of HIF (1), cats under related treatments were also excluded to avoid any potential influence of external EPO on biofluids HIF-1α concentrations.

Some limitations exist in the current study. Firstly, since cats with iron supplements or EPO-stimulating agents were excluded from the present research, the correlation between these therapies and biofluid HIF-1α and NTBI concentrations in CKD cats remains undetermined. Secondly, due to the retrospective nature of this study, not all the clinicopathological data are complete. These incomplete datasets make some statistical tests unfeasible and have relatively weakened the statistical power of our study. Thirdly, although we relied on the manufacturer’s validation data for the commercial ELISA kit, which was suitable for our study’s species, biofluid, and analyte range, independent validation was not performed. As this was an exploratory study, additional validation was not deemed necessary. However, in-house validation to confirm the robustness of the ELISA kit under similar conditions would be important in future studies. Lastly, age could certainly have a significant confounding effect on the results. However, due to the challenges of identifying healthy older cats without CKD in a veterinary teaching hospital setting, obtaining age-matched data was not feasible for our study.

In conclusion, it is well known that HIF serves as an important transcription factor during hypoxia. The present study has demonstrated that, in cats, pHIF-1α concentrations rise during the early stages of CKD, but then gradually decrease as the severity of the CKD increases. In addition, uHIF-1α concentrations show a progressive decline as the CKD severity increases. In parallel, pHIF and uHIF concentrations show a positive correlation that is independent of renal function. When CKD-related prognostic factors like proteinuria are considered (33), uNTBI could be useful as a potential renal biomarker when evaluating CKD progression.

## Data Availability

The original contributions presented in the study are included in the article/Supplementary material, further inquiries can be directed to the corresponding author.

## References

[1] Schodel J Ratcliffe PJ. Mechanisms of hypoxia signalling: new implications for nephrology. Nat Rev Nephrol. (2019) 15:641–59. doi: 10.1038/s41581-019-0182-z, PMID: 31488900

[2] Ma C Wei J Zhan F Wang R Fu K Wan X. Urinary hypoxia-inducible factor-1alpha levels are associated with histologic chronicity changes and renal function in patients with lupus nephritis. Yonsei Med J. (2012) 53:587–92. doi: 10.3349/ymj.2012.53.3.587, PMID: 22477004 PMC3343432

[3] Movafagh S Raj D Sanaei-Ardekani M Bhatia D Vo K Mahmoudieh M. Hypoxia inducible factor 1: a urinary biomarker of kidney disease. Clin Transl Sci. (2017) 10:201–7. doi: 10.1111/cts.12445, PMID: 28181420 PMC5421733

[4] Pan SY Tsai PZ Chou YH Chang YT Chang FC Chiu YL. Kidney pericyte hypoxia-inducible factor regulates erythropoiesis but not kidney fibrosis. Kidney Int. (2021) 99:1354–68. doi: 10.1016/j.kint.2021.01.017, PMID: 33812664

[5] Lourenco BN Coleman AE Tarigo JL Berghaus RD Brown CA Rissi DR. Evaluation of profibrotic gene transcription in renal tissues from cats with naturally occurring chronic kidney disease. J Vet Intern Med. (2020) 34:1476–87. doi: 10.1111/jvim.15801, PMID: 32468592 PMC7379026

[6] Lourenco BN Coleman AE Schmiedt CW Brown CA Rissi DR Stanton JB. Profibrotic gene transcription in renal tissues from cats with ischemia-induced chronic kidney disease. Am J Vet Res. (2020) 81:180–9. doi: 10.2460/ajvr.81.2.180, PMID: 31985291

[7] van Swelm RPL Wetzels JFM Swinkels DW. The multifaceted role of iron in renal health and disease. Nat Rev Nephrol. (2020) 16:77–98. doi: 10.1038/s41581-019-0197-5, PMID: 31554933

[8] Agarwal AK. Iron metabolism and management: focus on chronic kidney disease. Kidney Int Suppl. (2021) 11:46–58. doi: 10.1016/j.kisu.2020.12.003, PMID: 33777495 PMC7983022

[9] Martines AM Masereeuw R Tjalsma H Hoenderop JG Wetzels JF Swinkels DW. Iron metabolism in the pathogenesis of iron-induced kidney injury. Nat Rev Nephrol. (2013) 9:385–98. doi: 10.1038/nrneph.2013.98, PMID: 23670084

[10] Breuer W Ronson A Slotki IN Abramov A Hershko C Cabantchik ZI. The assessment of serum nontransferrin-bound iron in chelation therapy and iron supplementation. Blood. (2000) 95:2975–82. doi: 10.1182/blood.V95.9.2975.009k03_2975_2982, PMID: 10779448

[11] Esposito BP Breuer W Slotki I Cabantchik ZI. Labile iron in parenteral iron formulations and its potential for generating plasma nontransferrin-bound iron in dialysis patients. Eur J Clin Investig. (2002) 32:42–9. doi: 10.1046/j.1365-2362.2002.0320s1042.x, PMID: 11886431

[12] Prakash M Upadhya S Prabhu R. Serum non-transferrin bound iron in hemodialysis patients not receiving intravenous iron. Clin Chim Acta. (2005) 360:194–8. doi: 10.1016/j.cccn.2005.04.024, PMID: 15979061

[13] Hirota K. An intimate crosstalk between iron homeostasis and oxygen metabolism regulated by the hypoxia-inducible factors (HIFs). Free Radic Biol Med. (2019) 133:118–29. doi: 10.1016/j.freeradbiomed.2018.07.018, PMID: 30053508

[14] Haase VH. Hypoxia-inducible factor-prolyl hydroxylase inhibitors in the treatment of anemia of chronic kidney disease. Kidney Int Suppl. (2021) 11:8–25. doi: 10.1016/j.kisu.2020.12.002, PMID: 33777492 PMC7983025

[15] Chen N Hao C Peng X Lin H Yin A Hao L. Roxadustat for Anemia in patients with kidney disease not receiving Dialysis. N Engl J Med. (2019) 381:1001–10. doi: 10.1056/NEJMoa1813599, PMID: 31340089

[16] Chen N Hao C Liu BC Lin H Wang C Xing C. Roxadustat treatment for Anemia in patients undergoing long-term Dialysis. N Engl J Med. (2019) 381:1011–22. doi: 10.1056/NEJMoa1901713, PMID: 31340116

[17] Charles S Sussenberger R Settje T Langston C Lainesse C. Use of molidustat, a hypoxia-inducible factor prolyl hydroxylase inhibitor, in chronic kidney disease-associated anemia in cats. J Vet Intern Med. (2024) 38:197–204. doi: 10.1111/jvim.16807, PMID: 37740521 PMC10800191

[18] Matta MK Beekman CR Gandhi A Narayanasamy S Thomas CD Mohammad A. Determination of non-transferrin bound Iron, transferrin bound Iron, drug bound Iron and Total Iron in serum in a rats after IV Administration of Sodium Ferric Gluconate Complex by simple ultrafiltration inductively coupled plasma mass spectrometric detection. Nanomaterials. (2018) 8:101. doi: 10.3390/nano8020101 29439469 PMC5853732

[19] Shao Y Lv C Yuan Q Wang Q. Levels of serum 25(OH)VD3, HIF-1α, VEGF, vWf, and IGF-1 and their correlation in type 2 diabetes patients with different urine albumin creatinine ratio. J Diabetes Res. (2016) 2016:1–7. doi: 10.1155/2016/1925424, PMID: 27069929 PMC4812448

[20] Kocyigit I Taheri S Eroglu E Sener EF Zararsiz G Uzun I. Systemic succinate, hypoxia-inducible Factor-1 alpha, and IL-1beta gene expression in autosomal dominant polycystic kidney disease with and without hypertension. Cardiorenal Med. (2019) 9:370–81. doi: 10.1159/000500478, PMID: 31319406

[21] Group KCW. KDIGO 2024 clinical practice guideline for the evaluation and Management of Chronic Kidney Disease. Kidney Int. (2024) 105:S117–s314. doi: 10.1016/j.kint.2023.10.018, PMID: 38490803

[22] Chiang CK Tanaka T Inagi R Fujita T Nangaku M. Indoxyl sulfate, a representative uremic toxin, suppresses erythropoietin production in a HIF-dependent manner. Lab Investig. (2011) 91:1564–71. doi: 10.1038/labinvest.2011.114, PMID: 21863063

[23] Noh H Yu MR Kim HJ Jeon JS Kwon SH Jin SY. Uremia induces functional incompetence of bone marrow-derived stromal cells. Nephrol Dial Transplant. (2012) 27:218–25. doi: 10.1093/ndt/gfr267, PMID: 21622994

[24] Bajema IM Wilhelmus S Alpers CE Bruijn JA Colvin RB Cook HT. Revision of the International Society of Nephrology/Renal Pathology Society classification for lupus nephritis: clarification of definitions, and modified National Institutes of Health activity and chronicity indices. Kidney Int. (2018) 93:789–96. doi: 10.1016/j.kint.2017.11.023, PMID: 29459092

[25] Brown CA Elliott J Schmiedt CW Brown SA. Chronic kidney disease in aged cats: clinical features, morphology, and proposed pathogeneses. Vet Pathol. (2016) 53:309–26. doi: 10.1177/0300985815622975, PMID: 26869151

[26] McLeland SM Cianciolo RE Duncan CG Quimby JM. A comparison of biochemical and histopathologic staging in cats with chronic kidney disease. Vet Pathol. (2015) 52:524–34. doi: 10.1177/0300985814561095, PMID: 25516066

[27] Hokamp JA Leidy SA Gaynanova I Cianciolo RE Nabity MB. Correlation of electrophoretic urine protein banding patterns with severity of renal damage in dogs with proteinuric chronic kidney disease. Vet Clin Pathol. (2018) 47:425–34. doi: 10.1111/vcp.12648, PMID: 30125968

[28] Wang GL Semenza GL. Purification and characterization of hypoxia-inducible factor 1. J Biol Chem. (1995) 270:1230–7. doi: 10.1074/jbc.270.3.1230, PMID: 7836384

[29] van Raaij S van Swelm R Bouman K Cliteur M van den Heuvel MC Pertijs J. Tubular iron deposition and iron handling proteins in human healthy kidney and chronic kidney disease. Sci Rep. (2018) 8:9353. doi: 10.1038/s41598-018-27107-8, PMID: 29921869 PMC6008459

[30] Brown EA Sampson B Muller BR Curtis JR. Urinary iron loss in the nephrotic syndrome--an unusual cause of iron deficiency with a note on urinary copper losses. Postgrad Med J. (1984) 60:125–8. doi: 10.1136/pgmj.60.700.125, PMID: 6709543 PMC2417690

[31] Howard RL Buddington B Alfrey AC. Urinary albumin, transferrin and iron excretion in diabetic patients. Kidney Int. (1991) 40:923–6. doi: 10.1038/ki.1991.295, PMID: 1762297

[32] Nakatani S Nakatani A Ishimura E Toi N Tsuda A Mori K. Urinary Iron excretion is associated with urinary full-length Megalin and renal oxidative stress in chronic kidney disease. Kidney Blood Press Res. (2018) 43:458–70. doi: 10.1159/000488470, PMID: 29590662

[33] Vaden SL Elliott J. Management of Proteinuria in dogs and cats with chronic kidney disease. Vet Clin North Am Small Anim Pract. (2016) 46:1115–30. doi: 10.1016/j.cvsm.2016.06.009, PMID: 27485278

