# Supplementary material for: Evaluation of hypoxia-inducible factor-1α and urine non-transferrin-bound iron concentrations in cats with chronic kidney disease
Source: Front Vet Sci. 2024 Dec 19;11:1482998. doi: 10.3389/fvets.2024.1482998 (PMC11694447; doi:10.3389/fvets.2024.1482998)
Supplement: Supplementary file 1 [file Supplementary_Table_1.DOCX]

**Table S1: The precision of cat HIF-1α ELISA kit**

|  | Intra-assay precision | | | Inter-assay precision | | |
| --- | --- | --- | --- | --- | --- | --- |
| n | 20 | 20 | 20 | 6 | 6 | 6 |
| mean (pg/ml) | 12.84 | 49.32 | 190.44 | 10.60 | 44.84 | 184.72 |
| SD | 0.80 | 1.48 | 6.67 | 0.50 | 1.57 | 8.31 |
| CV (%) | 6.2 | 3.0 | 3.5 | 4.7 | 3.5 | 4.5 |

According to the manual, the sensitivity of the kit is 1.0 pg/ml. The detection range of the kit is 6.25-200 pg/ml, while the standard concentration gradients are composed of 6.25, 12.5, 25, 50, 100, and 200 pg/ml. Regarding the precision, samples in different concentrations of HIF-1α were tested 20 times in the same assay for the intra-assay CV, while samples were tested in 6 separate assays to assess the inter-assay CV.

**Validations of NTBI concentration with ICP-MS**

1. **Limit of Detection (LOD), Limit of Quantification (LOQ)**

In nature, it was unattainable to make the blank samples contain the same matrix as the test samples, so the procedural blank, replacing the serum/urine samples with the ultrapure water and undergoing the same sample preparation steps mentioned above, was applied to detect the LOD and the LOQ. The procedural blank was analyzed 5 times to obtain the mean of the signals (Sd) and the standard deviation of the signals (σ). The LOD and the LOQ were calculated as Sb+3σ and Sb+10σ, respectively. The LOD and LOQ were 5.12 ppb and 6.25 ppb, respectively.

1. **Working Range**

The stock solution containing 100 ng/ml of iron was diluted in linearity (5, 10, 15, 20, 25, 30, 35, 40, 45, and 50 ng/ml) via 0.0049M magnesium chloride with 2% nitric acid, which also served as 0 ng/ml.

**Figure S1: The calibration curve of ^57^Fe**

1. **Quality Control (QC)**

For the calibration curve, the coefficient of determination (r2) should be ≥ 0.995. Meanwhile, the range of relative error should be +/- 10%. Regarding the QC sample recheck, three spikes in different concentrations were selected, and the concentrations were detected between every 10 samples. Meantime, the relative error and the range of relative error should be +/- 20%.

**Table S2: The QC sample recheck by ICP-MS**

| Spike | 5 ppb | 15 ppb | 50 ppb |
| --- | --- | --- | --- |
| n | 5 | 5 | 5 |
| mean (ppb) | 5.18 | 14.58 | 50.36 |
| SD | 0.30 | 0.43 | 0.98 |
| CV (%) | 5.81 | 2.92 | 1.95 |

1. **Accuracy**

No certified reference materials of the feline NTBI could be applied for accuracy determination.

1. **Precision**

Three feline serum/urine samples in different NTBI concentrations were selected for the validation of inter- and intra-day coefficients of variability (CV). The inter-CV was accessed on three different days, and the intra-CV was analyzed three times within the same day.

**Table S3: The precision of the serum samples**

|  | Intra-day precision | | | Inter-day precision | | |
| --- | --- | --- | --- | --- | --- | --- |
| n | 3 | 3 | 3 | 3 | 3 | 3 |
| mean (ppb) | 9.57 | 14.71 | 25.70 | 8.96 | 12.44 | 21.30 |
| SD | 0.16 | 0.08 | 0.58 | 0.43 | 1.04 | 0.79 |
| CV (%) | 1.67 | 0.54 | 2.26 | 4.77 | 8.37 | 3.72 |

**Table S4: The precision of the urine samples**

|  | Intra-day precision | | | Inter-day precision | | |
| --- | --- | --- | --- | --- | --- | --- |
| n | 3 | 3 | 3 | 3 | 3 | 3 |
| mean (ppb) | 13.03 | 24.75 | 50.29 | 9.11 | 13.89 | 29.09 |
| SD | 0.26 | 0.42 | 1.13 | 0.44 | 1.21 | 0.89 |
| CV (%) | 1.97 | 1.69 | 2.25 | 4.78 | 8.71 | 3.06 |

1. **Bias-Matrix Effect**

The concentrations of NTBI in one feline serum/urine sample, three spikes in different NTBI concentrations, and the spiked sample (the volume of the sample and the spike was 1:1) were detected and calculated for the recovery rate.

**Table S5: The recovery of the bias-matrix effect in serum samples**

| Measured spike concentration (ppb) | Measured sample concentration (ppb) | Measured spiked sample concentration (ppb) | Expected spiked sample concentration (ppb) | Recovery (%) |
| --- | --- | --- | --- | --- |
| 4.64 | 16.30 | 10.87 | 10.47 | 103.82 |
| 14.93 | 16.30 | 16.63 | 15.62 | 106.47 |
| 25.30 | 16.30 | 20.02 | 20.80 | 96.25 |

**Table S6: The recovery of the bias-matrix effect in urine samples**

| Measured spike concentration (ppb) | Measured sample concentration (ppb) | Measured spiked sample concentration (ppb) | Expected spiked sample concentration (ppb) | Recovery (%) |
| --- | --- | --- | --- | --- |
| 9.36 | 32.54 | 20.60 | 20.95 | 98.33 |
| 20.35 | 32.54 | 25.94 | 26.45 | 98.07 |
| 30.05 | 32.54 | 30.19 | 31.30 | 96.45 |

1. **Dilution Recovery**

Three feline serum/urine samples in different NTBI concentrations were chosen and diluted serially into 1:2 and 1:4 via 0.0049M magnesium chloride with 2% nitric acid. These processed samples were analyzed and calculated for the recovery rate.

**Table S7: The recovery of dilution in serum samples**

| Measured sample concentration (ppb) | Dilution ratio | Expected measured concentration (ppb) | Observed measured concentration (ppb) | Recovery (%) |
| --- | --- | --- | --- | --- |
| 11.45 | 1:2 | 5.73 | 5.21 | 90.92 |
| 11.45 | 1:4 | 2.86 | 2.96 | 103.50 |

**Table S8: The recovery of dilution in serum samples**

| Measured sample concentration (ppb) | Dilution ratio | Expected measured concentration (ppb) | Observed measured concentration (ppb) | Recovery (%) |
| --- | --- | --- | --- | --- |
| 26.24 | 1:2 | 13.12 | 14.42 | 109.91 |
| 26.24 | 1:4 | 6.56 | 7.05 | 107.47 |

**Table S9: The operating condition of the ICP-MS**

| **Mass spectrometer parameters** | |
| --- | --- |
| Resolution | <0.8 amu at 10% peak maximum |
| Dwell time | 0.3 seconds |
| Peak pattern | 3 points |
| Sweeps per reading | 50 |
| Readings | 1 |
| Replicates | 3 |
| Ion lenses model | x-Lens |
| Isotopes monitored | ^57^Fe |
| **ICP parameters** | |
| Nebulizer type | MicroMist |
| RF power | 1550 W |
| Plasma gas flow rate | 14.99 L/min |
| Auxiliary gas flow rate | 0.902 L/min |
| Carrier gas | 1.01 L/min |
| Nebulizer pump (stabilize) | 0.1 rps |
| Nebulizer pump (rinse, uptake) | 0.3 rps |
| Sampler cones | Platinum/Nickel |
| Skimmer cones | Platinum/Nickel |
| Uptake time | 30 seconds |
| Stabilizing time | 30 seconds |
| Rinse time | 40 seconds |
| Rinse solution | 2% HNO_3_ |
| Use gas | Ar |
